# Hirsutism Caused by Pregnancy Luteoma in a Low-Resource Setting: A Case Report and Literature Review

**DOI:** 10.1155/2021/6695117

**Published:** 2021-03-24

**Authors:** David Hamisi Mvunta, Fatemazahra Amiji, Mubina Suleiman, Francisco Baraka, Ikrah Abdallah, Mabula Kazabula, Peter J. T. Wangwe, Furaha August

**Affiliations:** ^1^Department of Obstetrics and Gynecology, Muhimbili University of Health and Allied Sciences, 9 United Nations Road, Upanga West, P.O. Box 65017, Dar es Salaam, Tanzania; ^2^Department of Obstetrics and Gynecology, Mawenzi Regional Referral Hospital, P.O. Box 3054, Moshi, Tanzania; ^3^Department of Obstetrics and Gynecology, Mnazi Mmoja Hospital, P.O. Box 236, Zanzibar, Tanzania; ^4^Department of Obstetrics and Gynecology, Maweni Regional Referral Hospital, P.O. Box 16, Kigoma, Tanzania; ^5^Department of Obstetrics and Gynecology, Kondoa District Hospital, P.O. Box 40, Dodoma, Tanzania; ^6^Department of Obstetrics and Gynecology, Lugalo Military Hospital, P.O. Box 60126, Mwenge, Dar es Salaam, Tanzania

## Abstract

**Background:**

Pregnancy luteomas are rare, benign, ovarian neoplasms resulting from increased androgenic activity during pregnancy. Often, they occur asymptomatically and are only diagnosed incidentally during imaging or surgery: cesarean section or postpartum tubal ligation. Most common symptoms associated with pregnancy luteoma include acne, deepening of voice, hirsutism, and clitoromegaly. Most pregnancy luteomas regress spontaneously postpartum. Thus, the management of pregnancy luteomas depends on the clinical situation.

**Case:**

We report a case of 28-year-old gravida 2, para 1 who presented at 39 + 1 weeks of gestation with prolonged labor and delivered by emergency cesarean. Intraoperatively, a huge left ovarian mass was identified and resected, and tissue was sent for histopathology and a diagnosis of pregnancy luteoma was made after the pathological report.

**Conclusion:**

The present report emphasizes that pregnancy luteoma is a benign neoplasm and imprudent surgical intervention should be reserved. Proper imaging techniques, preferably MRI or ultrasonography that visualize the size of the ovary and reproductive hormonal profiles, would suffice for the diagnosis and management of pregnancy luteoma.

## 1. Introduction

Pregnancy luteoma is a rare nonneoplastic tumor-like lesion of the ovary that has an increased androgenic activity during pregnancy [1]. It is associated with varied symptoms, for instance, hirsutism, acne, deepening of voice, and virilization symptoms [2]. The first occurrence was reported by Sternberg and Barclay in 1966, and to date, very few cases have been documented in the literature [3]. It is almost always incidentally identified during operation, either at the time of cesarean section or during postpartum tubal ligation [3]. Many cases have been documented to resolve postpartum, but some have been associated with recurrence in the subsequent pregnancy [4]. Accurate case diagnosis is pertinent to avoid unnecessary oophorectomy, which may result in grave effects in the subsequent years. We present a woman incidentally found to have a large solid ovarian mass that presented a diagnostic dilemma intraoperatively and confirmed only after a pathological report.

## 2. Case Report

A 28-year-old pregnant woman with BMI > 30, gravida 2, para 1, at 39 weeks and 1 day of gestation, was admitted to the antenatal ward presenting with lower abdominal pain and mucoid bloody stained discharge. Her current antenatal, medical, and gynecological history was unremarkable; menarche was during her 10^th^ year; and her menses were painless and of regular length, amount, and flow. During the index pregnancy, nothing remarkable was noted during her antenatal visits. She did two obstetric ultrasounds at 31 and 37 weeks, and both showed a viable fetus with no adnexal masses.

Past obstetric history is remarkable for preeclampsia from the 24^th^ week of pregnancy and vaginal delivery of a premature male stillbirth of 1.5 kg about 2 years ago. She acknowledged having facial hirsutism that started from the 20^th^ week of pregnancy and persisted postpartum but was not associated with deepening of the voice or other virilization symptoms.

On general examination, she had normal findings, with normal female hair distribution except for facial hair at the chin, and no other signs of virilization were noted. She reported that acne was present earlier in pregnancy but subsided as the pregnancy progressed.

### 2.1. Labor and Delivery

**Figure 1:**
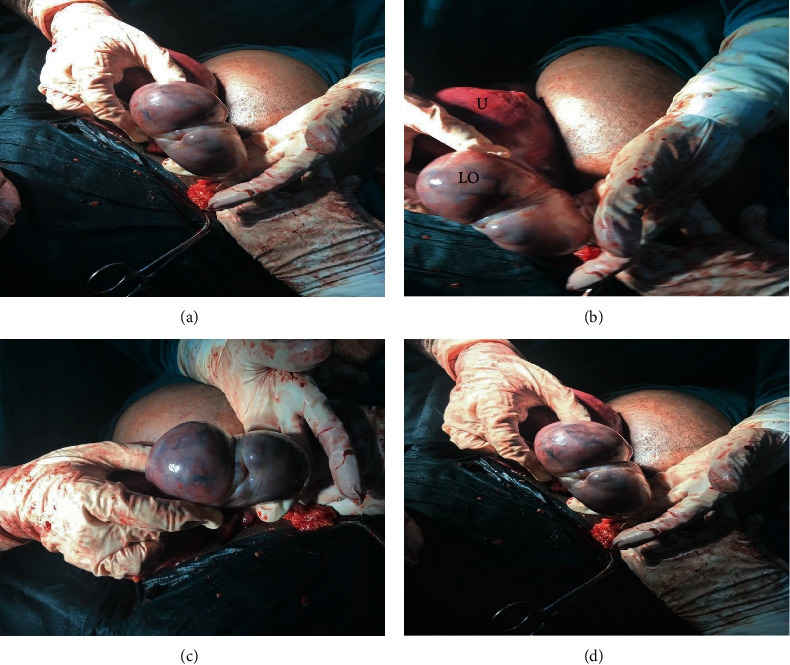
(a–d) Photographs showing grossly enlarged left ovary. (b) Demonstrates the relations of the enlarged left ovary (LO) to the uterus (U).

**Figure 2:**
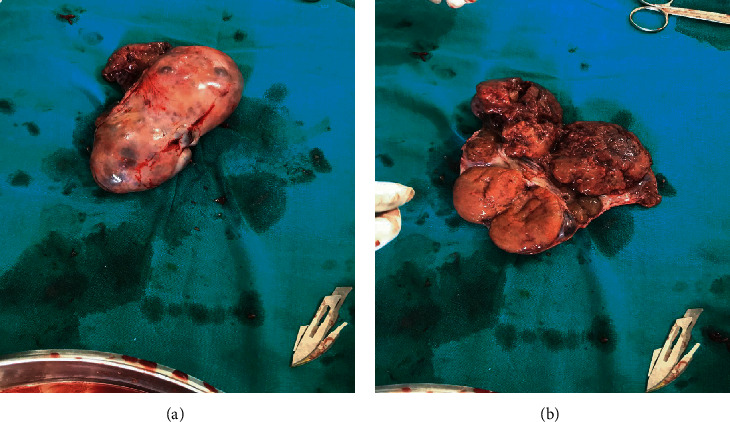
(a, b) A whole section of the enlarged left ovary after unilateral oophorectomy.

Her antenatal work-up revealed she was in the latent phase of labor and was kept for observation. Following an active onset of labor, she was transferred to the labor ward, but her labor progressed poorly and was prepared for emergency cesarean. Intraoperatively, a healthy female infant (with no virilization features) weighing 3.5 kg with Apgar scores of 9 and 10 at the first and fifth minutes, respectively, was extracted, and the uterus was then repaired accordingly. During the repair, a huge mass on the left adnexa measuring about 9 cm × 7 cm × 5.5 cm involving the left ovary was noted (Figures 1(a)–1(d)), and unilateral oophorectomy was performed. The whole ovarian tissue (Figures 2(a) and 2(b)) was then sent for permanent section histopathological assessment suspecting ovarian malignancy. The uterus and right ovary both appeared macroscopically normal.

**Figure 3:**
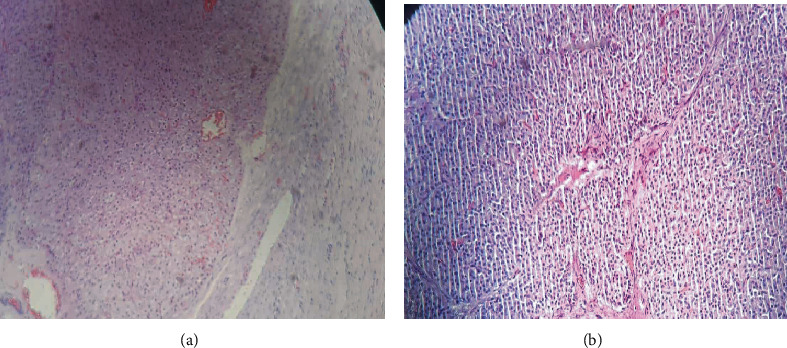
(a, b) Microscopic view of ovarian tissue after H and E stain.

The gross pathological examination revealed a large ovarian tissue with two grossly visible yellowish tumor-like marked granular cysts measuring 1.5 cm by 1 cm and 1 cm by 1 cm. On histology, there were granular luteinized cells in both cell masses but were otherwise normal ovarian tissues with hemorrhages and two follicular cysts with 2-3 mitoses/10 HPF (Figures 3(a) and 3(b)). Finally, it was diagnosed as pregnancy luteoma, with no obvious malignancy.

### 2.2. Literature Review

We reviewed various pregnancy luteoma case reports published in English between 2000 and 2020. Our review included studies obtained from reference to published articles and literature search engines: PubMed or Google Scholar, using the terms “pregnancy luteoma.” A total of 205 case reports were reviewed from PubMed and 2,100 from Google Scholar. We identified and summarized 24 articles reporting 25 cases of pregnancy luteoma [2, 4–25] (Table 1).

## 3. Discussion

Pregnancy luteoma is a rare benign tumor-like enlargement of the ovary accompanying pregnancy [4]. An occurrence preponderance has been noted among the Afro-Caribbean around the ages of 30 and 40 years old and those with preexisting PCOS [11]. PCOS contributes to high *β*-hCG hormone levels favoring the proliferation of pregnancy luteoma [2, 26]. These findings on age of occurrence are consistent with our case report. The present literature review adds evidence to this finding on age of occurrence (Table 1). In addition, we found the occurrence not to be specific to the Afro-Caribbean but rather multiracial (Table 1). The exact age of incidence is unknown since most patients are asymptomatic and often incidentally diagnosed intraoperatively during cesarean or postoperative tubal ligation [4]. Furthermore, pregnancy luteoma masses have been reported to cause dystocia [7, 27], a finding that could have occurred in our case since C-section was done due to poor progress of labor. The etiology of pregnancy luteoma, although unclear, is proposed to arise from the proliferation of theca-lutein cells following hormonal stimulation [3].

Patients with pregnancy luteoma have been reported to present with features of androgen excess, for instance, acne, deepening of the voice, facial and/or abdominal hirsutism, and clitoromegaly [4]. These symptoms have been reported to begin sometime during pregnancy and subside or stop during the postpartum period (Table 1). Unfortunately, in other patients, not all of the symptoms subside: hirsutism, deepening of the voice, and clitoromegaly seem to persist while acne and hair loss subside [4]. In the present case report, the time for follow-up was short to ascertain whether there was subsiding of the mentioned symptom of hirsutism. This is a limitation in our case report. Several authors have shown virilization symptoms in the female fetus [11], a finding that was absent in our case report. It is hypothesized that despite the maternal hyperandrogenemia produced by the pregnancy luteoma between the critical 9-14 weeks of development, the placenta is somewhat protective against the masculinization of the female fetus by converting the excess androgens to estrogens [5]. Emerging evidence has implicated androgenemia, specifically elevated testosterone, as a causative for preeclampsia [28]. Furthermore, preeclamptic women have been reported to have high placental expressions of the androgen receptor (AR) gene and elevated levels of testosterone two- to threefolds compared non-preeclamptic women [28]. A finding that was consistent with our case report, she had hirsutism and preeclampsia. The only limitation in our case report was we failed to analyze the level of testosterone.

The diagnosis of pregnancy luteoma requires a high index of suspicion; once you suspect it based on the above-mentioned signs, perform detailed obstetric ultrasonography to visualize the size of the ovary. In addition, supportive investigations like hormonal assays should also be done to rule out other ovarian neoplasms [29].The management of pregnancy luteoma is case dependent (Table 1), for instance, cases presenting with severe virilization symptoms [2], pressure symptoms, or torsion will require prompt surgery while the asymptomatic cases require conservative managements with regular follow-up [30] as they usually regress spontaneously following delivery. Despite lacking hormonal assays in this case, spontaneous regression of hyperandrogenemia is the natural course of pregnancy luteoma [5]. Based on the presently described case, surgery for pregnancy luteoma was not indicated since there were no distressed symptoms. Instead, proper imaging techniques, preferably ultrasound or MRI and exploration of hormonal profile would suffice for diagnosis and management.

## 4. Conclusions

Based on the presently described case, pregnancy luteoma is a benign neoplasm and imprudent surgical intervention should be reserved. A high index of suspicion is paramount to diagnose pregnancy luteoma, and once suspected, one should perform a detailed obstetric ultrasound visualizing the size of the ovary and hormonal assay for testosterone and its derivatives.

## Figures and Tables

**Table 1:** Literature review of pregnancy luteoma case reports: from 2000 to 2020.

Case #	Patient	Author (year)	Presenting symptom & investigations	Management offered	Outcome (fetal genitalia effects)	Country (race)
1	28 yr old, G2P1, GA 39 w	Our case report	(1) Incidental operative finding with hirsutism	Cesarean (unrelated to luteoma)+left oophorectomy	Female infant (nil)	Tanzania (African)
			(2) USS-done but not seen, hormonal assays-not done			

2	25 yr old, G1P0, GA 28 w	Rapisarda et al. (2016)	(1) Facial acne, abdominal & facial hirsutism start GA 23 w	Conservative: till worsened clinical situation and cesarean @ 34 w GA+right oophorectomy	Male infant (nil)	Italy (Spanish)
			(2) USS-right adnexal mass, elevated male hormones (testosterone, DHEAS, SHBG, androstenedione			

3	28 yr old, G1P0, GA 28 w	Masarie et al. (2010)	(1) Incidental finding on 33 w USS, virilization	Cesarean @ 37 w	Female infant (nil)	USA (Latina)
			(2) Elevated testosterone			

4	G1P0	Wang et al. (2005)	(1) Dysuria, left flank pain, fever @ 35 w	Conservative: vaginal delivery @ 36 w	Female infant+virilization (clitoromegaly)	Taiwan
			(2) Imaging-bilateral adnexa masses+hydronephrosis, elevated testosterone			

5	26 yr old, G1P0, GA 35 w	Kao et al. (2005)	(1) Deepened voice, hirsutism, Elevated testosterone	Conservative: vaginal delivery @ 36 w	Female infant+virilization (clitoromegaly)	Taiwan
			(2) USS & MRI-bilateral ovarian enlargement	3^rd^ week postpartum, testosterone normalized; 2 months later, ovarian masses normalized, hirsutism improved but fetal clitoromegaly persisted		

6	28 yr old, full-term	Kumar et al. (2014)	(1) Incidental operative finding	Cesarean+right salpingo-oophorectomy	Fetus (nil)	India
			(2) USS-enlarged left ovary, hormonal studies-not done			

7	23 yr old, G1P0, GA 22 w	Tannus et al. (2009)	(1) Incidental USS finding @ 22 w	Conservative: induction of labor due to postdate; following failed induction cesarean+right oophorectomy	Male infant (nil)	USA
			(2) USS & MRI-right ovarian mass			

8	32 yr old, G2P0, GA 32 w	Dahl et al. (2008)	(1) Deepened voice @ 32 w, balding, clitoromegaly, hirsutism	Conservative to 36 w and cesarean (unrelated to luteoma)	Male infant	USA
			(2) Elevated testosterone			
			(3) USS & MRI-ovaries not visualized	Ovaries were reserved, developed lactational delay of 1 w		

9	21 yr old, 29 yr old, G1P0, GA 34 w	Nanda et al. (2014)	(1) Incidental USS finding-bilateral ovarian masses	Cesarean (unrelated to luteoma)+bilateral oophorectomy	Female infant (nil)	Oman

10	30 yr old, amenorrhoeic for 2 months	Brar et al. (2017)	(1) Feature suggestive of ectopic pregnancy (abdominal pain, vomiting)	Explorative laparotomy for raptured ectopic pregnancy+salpingo-oophorectomy	—	India
			(2) USS-raptured tubal ectopic pregnancy, solid right ovarian mass			

11	28 yr old, G1P0, GA 42 w	Roth et al. (2000)	Incidental operative finding	Cesarean after failed induction	Infant with ambiguous genitalia+virilization (clitoromegaly)	German

12	36 yr old, G1P0, conceived following IVF	Spitzer et al. (2007)	(1) Features of GDM, gestational HTN	Assisted vaginal delivery (unrelated to luteoma) @ 36 w	Female infant with ambiguous	USA
			(2) USS-solid lesions suggesting fibroids (from 6^th^ to 29^th^ w GA)	On postpartum D-18 laparotomy+omentectomy+right salpingo-oophorectomy done	Genitalia	
			(3) Review of maternal hx: acne, deepening of voice, hirsutism, clitoromegaly	(prenatal fibroids not seen, probably were enlarged ovaries)		
			(4) Labs; elevated testosterone			
			(5) USS on 12^th^ postpartum day-complex right ovary	Frozen sections suggested stromal tumor, but final pathological report confirmed luteoma		

13	39 yr old, G2P1, hx of primary subfertility and underwent a wedge resection of left ovary for PCOD	Banerjee et al. (2006)	(1) Uneventful during with episodes of threatened miscarriage and pre-eclampsia, sickle cell gene carrier	Cesarean+left cystectomy	Male infant	UK (black)
			(2) USS @ 12^th^ & 20^th^ w GA-no adnexal masses			
			(3) Incidental operative finding			

14	33 yr old, G1P0, GA 35 w	Ugaki et al. (2009)	(1) Retrospectively study of hx, hair loss, hirsutism, deepening of voice	Cesarean+left cystectomy	Female infant+virilization (clitoromegaly)	Japan
			(2) TUSS-left ovarian tumor			

15	33 yr old, G1P0, GA 33 w	Tan et al. (2008)	(1) Features of raptured ovarian torsion: severe abdominal pain and decreasing Hb @ GA 33 w and treated but recurred after delivery @ 36 w GA	Diagnostic laparotomy+right salpingo-oophorectomy done	Not mentioned	Singapore (Indian)
			(2) USS-enlarged right adnexa mass probably ovarian with intratumoral bleeding			

16	>33 yr old, G2P1, GA 33 w	Choi et al. (2000)	(1) New onset hirsutism @ GA 28 w	Spontaneous preterm labor @ GA 29 w	Female infant	USA (Hispanic)
			(2) USS-enlarged right ovary			
			(3) elevated maternal testosterone			
			(1) Abnormal results of triple screen test	Surgery: left oophorectomy (luteoma of pregnancy) and right anterior mass identified as left lobe of liver		USA (white)
	>30 yr old, G2P1, GA 21 w		(2) USS-left adnexa mass with right anterior abnormal mass	Pregnancy left in situ	Pregnancy ongoing at time case publication	

17	>33 yr old, G1P0, GA 19 w	Khurana et al. (2017)	(1) Incidental USS finding and follow up MRI-unilateral ovarian mass	Surgery: explorative laparotomy and left oophorectomy (dx after as luteoma of pregnancy) and pregnancy left in situ	A term baby at 40 weeks	USA

18	>28 yr old, G4P3, amenorrhoea 2/12	Rathore et al. (2017)	(1) Presented with signs of ectopic pregnancy and USS showed tuboovarian mass	Surgery: emergency laparotomy and salpingo-oophorectomy	Not applicable	India

19	>40 yr old, G2P1, GA 16 w	Wadzinski et al. (2014)	(1) Increasing acne, facial hair, and deepening of voice	Surgery: C-section of twins @ 33 w of GA due to preeclampsia	Twins, female infants with ambiguous genitalia	USA (Caucasian)
			(2) Lab: elevated testosterone			
			(3) USS: negative for mass			

20	>33 yr old, GA 17 w	Verma et al. (2016)	(1) Asymptomatic	Surgery: C-section and unilateral oophorectomy suspecting malignancy	Female infant	India
			(2) Incidentally identified intraoperatively			

21	>25 yr old, GA 37 w	Holt et al. (2005)	(1) Hair on face and abdomen, deepened voice	1^st^ pregnancy: SVD @ 38 w	Male infant	UK
			(2) USS done 4 w postpartum: enlarged ovaries			
			(3) 3 yr later on another pregnancy above symptoms recurred	2^nd^ pregnancy: SVD @ 39 w	Male infant	
			(4) Repeat USS done 5 w postpartum: normal sized ovaries			

22	>34 yr old, primigravida	Mazza et al. (2002)	(1) @ 5 k GA abdominal pain and USS: normal gestational sac with enlarged right ovary	Surgery: laparotomy and C-section (due to fetal distress and raptured membranes)	Female infant with ambiguous genitalia	Italy
			(2) @ 20 w: abdominal pain and USS: both ovaries enlarged			
			(3) Last 3/12 of pregnancy: increased abdominal pain, lower extremity hair, deepening of voice, and clitoromegaly			

23	>29 yr old, primigravida, PIH	Dhar et al. (2019)	Incidental operative finding	Surgery: emergency C-section due to fetal distress and a “fibroid like mass” was excised	Not mentioned	India

24	>26 yr old, primigravida @ 8 w GA	Dasari et al. (2013)	(1) Febrile for 2 w, abdominal distension 4/7	Spontaneous miscarriage @ 17 w due to cervical incompetence.	Male fetus	India
			(2) labs: testosterone was elevated 30 times. Other labs were normal or inconclusive			
			(3) USS; bilateral ovarian masses, moderate ascites and pleural effusion			
			(4) Laparoscopy: enlarged ovaries			
			(5) Frozen section suggested luteoma			
			(6) Maternal hirsutism was conspicuous @ 16 w			

Abbreviations: yr: year; GA: gestation age; MRI: magnetic resonance imaging; USS: ultrasound; TUSS: transvaginal ultrasound; @: at; nil: means no feminizing features; Labs: laboratory findings; +: means with or and; PCOD: polycystic ovarian disease; IVF: in vitro fertilization; PIH: pregnancy induced hypertension.
